# The *Phyllodonta latrata* (Guenée) species group in Costa Rica (Geometridae, Ennominae)

**DOI:** 10.3897/zookeys.421.7590

**Published:** 2014-06-27

**Authors:** J. Bolling Sullivan

**Affiliations:** 1 200 Craven St., Beaufort, North Carolina 28516 USA

**Keywords:** Geometridae, Ennominae, *Phyllodonta*, Costa Rica

## Abstract

Historically, the name *Phyllodonta latrata* (Guenée) has been applied to what is a complex of three undescribed species in Costa Rica. They are very similar in maculation, but can be differentiated by genitalic characters and barcodes. *P. alajuela* Sullivan, **sp. n.** occurs at lower altitudes in the northwestern part of Costa Rica whereas *P. intermediata* Sullivan, **sp. n.** and *P. esperanza* Sullivan, **sp. n.** are found at partially overlapping altitudes in the central mountain ranges.

## Introduction

The genus *Phyllodonta* Warren currently consists of approximately 26 species of medium-sized geometrid moths that occur from the southern United States to Argentina (Pitkin 2002). Currently, eight species are thought to occur in Costa Rica (Pitkin et al. 1996). Unpublished mtDNA barcode data indicate that many of these species are complexes of similar-looking species often with non-overlapping geographic ranges. *Phyllodonta latrata* (Guenée) was described from Brazil and Colombia; in the British Museum the specimen from Nova Friburgo (state of Rio de Janeiro, Brazil) is labeled as the type. It is unlikely that specimens from Colombia are conspecific. In Costa Rica, specimens that are very similar to *Phyllodonta latrata* occur from about 500–3000 m, but mtDNA barcode sequences of distinct clusters suggest a complex of species. Four additional species from Peru and Ecuador have been subject to barcode analysis but no haplotype clusters overlap those from Costa Rica. There are no specimens of *Phyllodonta latrata* from Brazil that have been barcoded nor have the types of *Phyllodonta latrata* nor *Phyllodonta succedens* (Walker) (TL: Quito, Ecuador and Bogota, Colombia) been dissected. Currently, *Phyllodonta succedens* is placed as a synonym of *Phyllodonta latrata* in the British Museum collections, although Pitkin (2002) regarded it as a valid species with *Phyllodonta nolckeniata* (Snellen) (TL: Bogota, Colombia) as a synonym.

## Materials and methods

Photographic methods used herein are described in Sullivan and Adams (2009). Procedures for dissecting and preparing genitalia follow that of Lafontaine (2004). DNA sequencing of the barcode fragment of the COI gene was carried out at the Canadian Center for DNA Barcoding in Guelph, Ontario. Barcode sequences were compared by nearest neighbor analyses as implemented on the Barcode of Life Data systems website (Ratnasingham and Hebert 2007).

### Repository abbreviations

BMNH Natural History Museum, London, UK

INBio Instituto Nacional de Biodiversidad, Santo Domingo de Heredia, Costa Rica

JBS J. Bolling Sullivan, Beaufort, North Carolina, USA

USNM National Museum of Natural History, Washington, District of Columbia, USA

## Systematics

I have photographed and examined the types of 26 of the 31 names applied to this genus. *Phyllodonta latrata* (Gn.), *Phyllodonta succedens* (Wlk.) and *Phyllodonta nolckeniata* (Snellen) are distinguished by their rich brown color and will be referred to as the *Phyllodonta latrata* complex. The antemedial and postmedial lines are usually distinct and are wavy or undulating. On the underside, these lines are distinct and the characteristic whitish blotch (Pitkin 2002) at the outer anal margin of the forewing is present with a blackish blotch proximal to it. The characteristic juxtal sac and socii (Pitkin 2002), unique to the genus, are present. *Phyllodonta catephracta* Prout is somewhat similarly colored, but has a distinct, wide submarginal line on the forewing not present in the *Phyllodonta latrata* group. *Phyllodonta peccataria* (Barnes & McDunnough) and *Phyllodonta sarukhani* Beutelspacher are similar in general color but the wing markings and genitalia are very different (Ferris and Walsh 2006). I have examined the genitalia of nine other taxa in the genus and the compact shape of the socii and maculation seem to distinguish the *Phyllodonta latrata* group. Given that barcodes of the *Phyllodonta latrata* group specimens from Ecuador and Peru do not match those of any of the Costa Rican species, and given that species of Southeastern Brazil rarely occur in Costa Rica, all of the species in Costa Rica are treated as new.

### Phyllodonta esperanza

Taxon classificationAnimaliaLepidopteraGeometridae

Sullivan sp. n.

http://zoobank.org/E9315F5A-D858-473C-8680-54323A1A371C

Figs 1 – 5 , 16

#### Type material.

Holotype male: Costa Rica, Tapanti Parque (9.456°N, 83.417°W), Cartago Province, 1275 m, 7–9 July 2008, J. Bolling Sullivan (DNA voucher #11-CRBS-381) (INBio). **Paratypes:** 5♂, 4♀: 1♂, same data as holotype (DNA voucher #11-CRBS-288); 1♂, same data as holotype but 12–17 February 2005 (07-CRBS-1206), (dissection #JBS-3305); 1♂, Costa Rica, Parque National Volcan Poas (10.103°N, 84.342°W), Alajuela Province, 2500 m, 7–8 August 2007, J. Bolling Sullivan (07-CRBS-359), (dissection #JBS-2006); 2♀, same data (07-CRBS-357), (JBS-2007); 3♂, Costa Rica, Villa Mills (9.334°N, 83.423°W), Cartago Province, 2845 m, J. Bolling Sullivan (10-CRBS-764, 767, 1531), 2♀, same data (10-CRBS-765, 766). BMNH, INBio, JBS, USNM.

#### Etymology.

The species is named for the biological station La Esperanza in the Talamanca Mountains, Cartago, Costa Rica, where many of the specimens were taken.

#### Diagnosis.

Maculation does not seem to be diagnostic for distinguishing this species. It is best characterized by barcode data and the genitalia. It has the highest altitude distribution but does overlap that of *Phyllodonta intermediata* below 2300 m. In the male, the base of the socius is usually swollen (straight in the other two species); the vesica is unarmed with pouches emanating from the left side (right side in *Phyllodonta intermediata*, armed in *Phyllodonta alajuela*). In the female, the collar on the ductus bursae is narrow and the signum on the corpus bursae is crescent shaped, often with a medial kink and with the anterior and posterior sides narrowly separated. In the other two species the collar is wider and the signa are more oval shaped, never kinked.

#### Description.

**Figures 1, 2.**
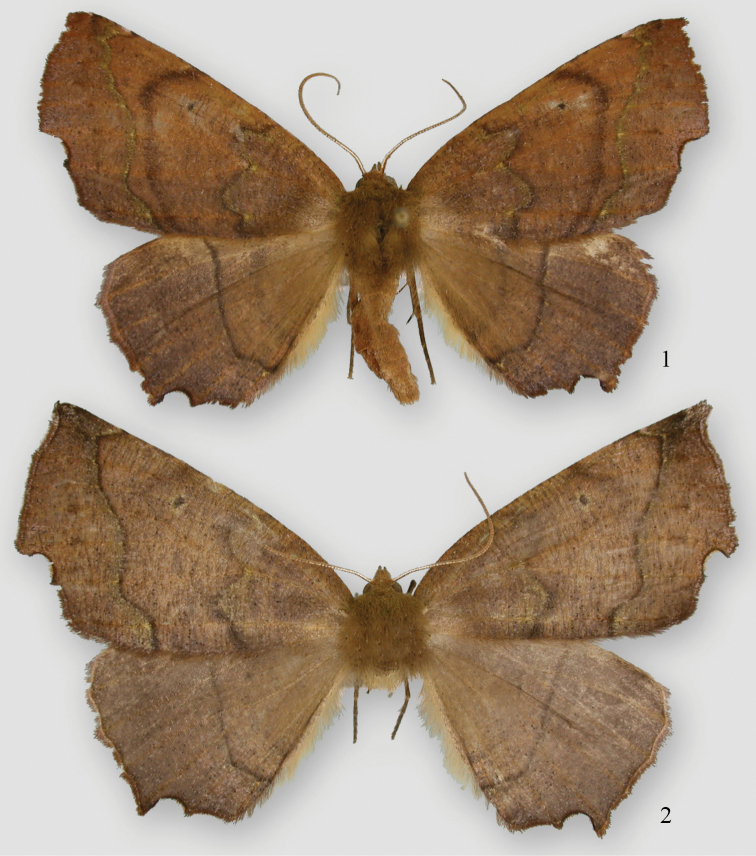
*Phyllodonta esperanza*. **1** male holotype (11-CRBS-381) **2** female (07-CRBS-359).

**Figures 3, 4.**
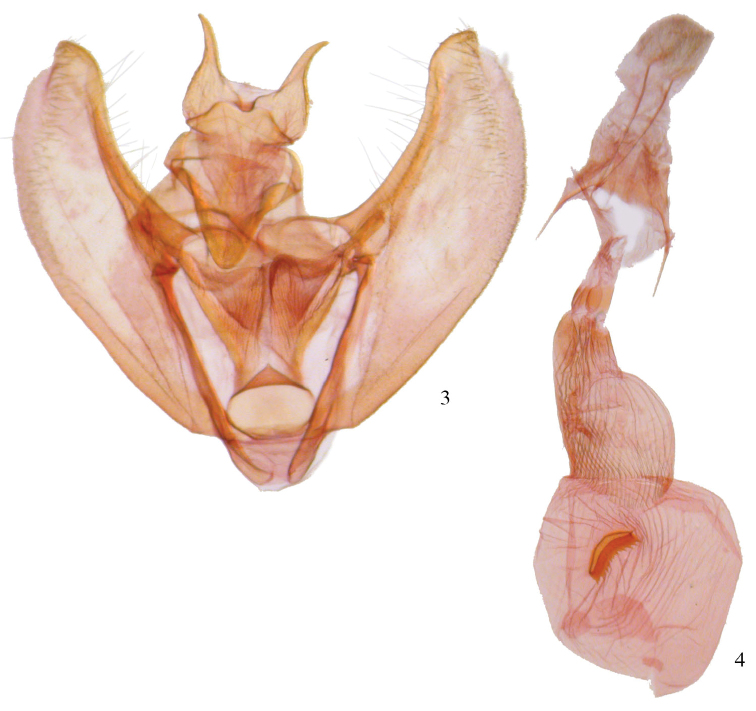
*Phyllodonta esperanza*. **3** male valve (JBS-1248) **4** female genitalia (JBS-2007).

**Figures 5, 6.**
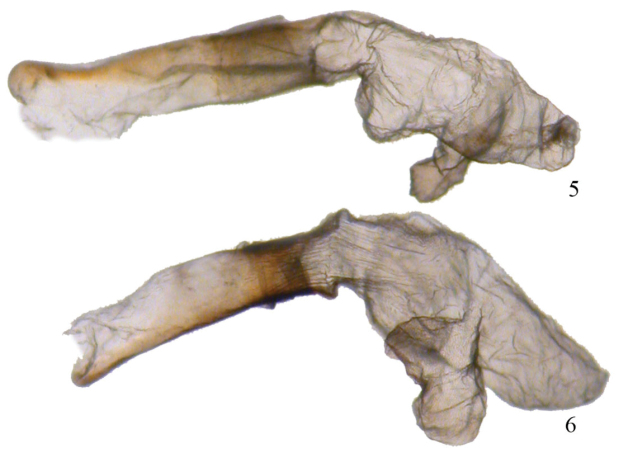
Aedeagi. **5** *Phyllodonta esperanza* **6** *Phyllodonta intermediata*.

**Male.** (Fig. 1) *Head*–labial palps warm brown, slightly porrect. First segment upcurved, second segment similar in length and tufted slightly over third segment, which is 1/3 as long and angled ventrally. Haustellum developed. Eyes hemispherical, large, ocellus present. Frons brown, slightly pointed with scaling directed anteriorly. Scape cream proximally, brown laterally and distally. Cream color extends along dorsal edge of antenna to tip. Antennal segments oblong with minute setae at base of each segment. Interantennal area warm brown, lighter onto collar. *Thorax and Abdomen*–Vertex brown, fine scaling extending onto thorax, most scales expanding to three pointed tip, a few black-tipped. Tegulae brown, abdomen with closely appressed scaling, gray brown dorsally, warm brown ventrally. Legs finely scaled, warm brown, tibia on first two legs with 3 evenly spaced white spots dorsally, proximal smallest, distal largest, tarsal spines prominent (0-2-4). *Wings*–forewings warm brown with prominent antemedial and postmedial lines that undulate, in both cases forming two outward bulges. Antemedial bulges separated by prominent cleft. Both lines black edged with lighter grayish scales proximally. Black scaling distal to reniform at costa and forming a diffuse line paralleling antemedial line. Reniform spot small, black and forming center of a gray circle. Forewings long, outer margin truncated at costa with a distinct notch medially, forewing length 23.0 mm (20–26 mm, n=18). Hindwing with prominent postmedial line, margin with submedial notch. Underside of wings warm brown, forewing with postmedial line prominent, medial line visible, antemedial line absent. White blotch subterminal at notch, larger black blotch proximal to it. Small discal spot present. Hindwing underside with prominent postmedial line, prominent discal spot, small yellow line of scales distal to postmedial line on both wings. Cream streak from wing base widening to anal angle. *Male Genitalia* (Figs 3, 5) (20 dissections) – uncus unsclerotized, short with terminal setae. Socius triangular shaped at base and curving distally toward tip. Well-defined tegumen with triangular gnathos, rounded at tip with small setae. Juxta narrow basally, forming a pocket and widening toward transitilla lobes that widen medially. Costa well sclerotized, tapering to tip of valva. Valva broad with two ridges bearing setae. Subcostal ridge tapers slightly and meets a broader submarginal ridge that broadens to half width at valve tip. Saccus V-shaped and rounded slightly at basal point. Aedeagus partially sclerotized distally, a band of striations at tip. Ductus inserts sub-basally. Vesica sac-like, curving ventrally; two outpockets medially, most prominent one bifurcated and curving to left with smaller lobe to right. No cornuti or sclerotized areas. Vesica slightly longer than aedeagus. **Female.** (Fig. 2) – similar to male but larger, gray-brown ground color, cross lines usually more prominent, forewing length 26.1 mm (25–27 mm, n=7). Gray circle around reniform more distinct. Undersides similar to male but gray brown. *Female Genitalia* (Figs 4, 16) (8 dissections) –Anal papillae with setae, distally truncated in shape with anterior apophyses shorter than posterior apophyses. Anal plate not sclerotized, forming a broad funnel. Ductus bursae moderately short with sclerotized collar at posterior end. Collar ends form obvious abutment. Accessory bursae striated, widening significantly prior to midpoint and striations continuing onto corpus bursae. Corpus bursae 2× as wide as ductus bursae, mostly unstriated with crescent-shaped signum. Signum varies but usually an elongate crescent often kinked medially and with several rows of broad basal spines on anterior side of crescent.

#### Barcodes.

Twenty nine specimens have been barcoded and exhibit twelve haplotypes that differ from each other by a maximum of 0.8%. They differ from those of *Phyllodonta intermediata* by a minimum of 4.6% and from *Phyllodonta alajuela* by a minimum of 4.8%. The most common haplotype (10-CRBS-762) is:

AACATTATATTTTATTTTTGGGATTTGAGCTGGAATAGTAGGAACATCTTTAAGTTTATTAATTCGAGCTGAATTAGGAAATCCTGGATCTCTAATTGGAGATGATCAAATTTATAATACTATTGTAACTGCTCATGCTTTTATTATAATTTTCTTTATAGTAATACCTATTATAATCGGAGGATTTGGAAATTGATTAGTTCCTTTAATATTAGGAGCTCCTGATATGGCTTTCCCTCGAATAAATAATATAAGATTTTGATTACTTCCACCTTCTATTACATTATTAATTTCTAGAAGAATTGTGGAAAATGGAGCTGGGACAGGATGAACTGTTTATCCTCCTTTATCTTCTAATATTGCTCACGGTGGTAGTTCTGTTGACCTTGCTATTTTTTCATTACATTTAGCTGGTATTTCATCAATTTTAGGGGCTATTAATTTTATTACTACAATTATTAATATACGATTAAATAATTTATCTTTTGATCAAATACCTTTATTTGTATGAGCAGTAGGAATTACTGCATTTTTATTATTATTATCATTACCTGTTTTAGCTGGAGCTATTACTATATTATTAACAGATCGAAATTTAAATACATCTTTTTTTGATCCTGCTGGAGGAGGAGATCCAATTTTATACCAACATTTATTT

#### Distribution.

Known from above 1200 m in the Talamancas as well as the Central Volcanic and Tilaran ranges in Costa Rica. In flight throughout the year.

#### Remarks.

Nothing is known about the biology of this species. Its range probably extends into the other mountain ranges in Costa Rican and perhaps in northern Panama.

### Phyllodonta intermediata

Taxon classificationAnimaliaLepidopteraGeometridae

Sullivan sp. n.

http://zoobank.org/FEE6C9E5-A097-4088-983C-D0573F1F0E1C

Figs 6 – 10 , 17

#### Holotype male.

Costa Rica, tunnel road, Tapanti Parque (9.43°N, 83.46°W) Cartago Province, 1475 m, 7–8 July 2008, J. Bolling Sullivan (10-CRBS-283) (INBio), **Paratypes.** 5♂, 1♀: 2♂, 1♀, same data as holotype (11-CRBS-2726, 10-CRBS-285 (JBS-2817), 10-CRBS-282 (JBS-5409)); 2♂, Costa Rica, Tapanti Parque (9.46°N, 83.42°W), Cartago Province, 1275 m, 7–9 July 2008, J. Bolling Sullivan, (11-CRBS-286 (JBS-5416); 1♂, Costa Rica, Vera Blanca, La Paz Waterfall Garden (10.12°N, 84.10°W), Alajuela Province, J. Bolling Sullivan (07-CRBS-1202), (JBS-3306) (INBio, JBS, USNM).

#### Etymology.

The species is named for the intermediate altitudes between 1200 and 1580 m where most of the specimens were taken. Two specimens were taken above 1600 m.

#### Diagnosis.

Maculation is not diagnostic for identifying this species. It is best characterized by barcode data and the genitalia. In the male, the distal side of the socius is usually straight (swollen in *Phyllodonta esperanza*), the vesica is unarmed and pouches emanate from the right side (in *Phyllodonta alajuela* the vesica is armed, in *Phyllodonta esperanza* the pouches emanate from the left side). In the female, the collar on the ductus bursae is narrow (broad in *Phyllodonta esperanza*)and the signum on the corpus bursae is peanut-shaped with moderate spacing between the anterior and posterior sides (crescent shaped and narrowly spaced sides in *Phyllodonta esperanza*, oval shaped in *Phyllodonta alajuela*).

#### Description.

**Figures 7, 8.**
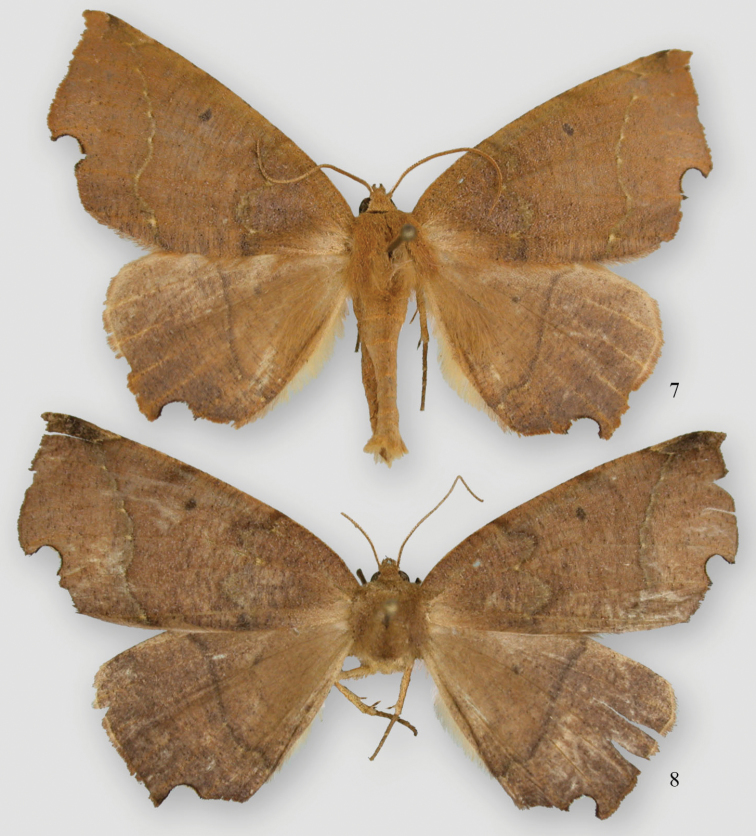
*Phyllodonta intermediata*. **7** male holotype (10-CRBS-283) **8** female (10-CRBS-282).

**Figures 9, 10.**
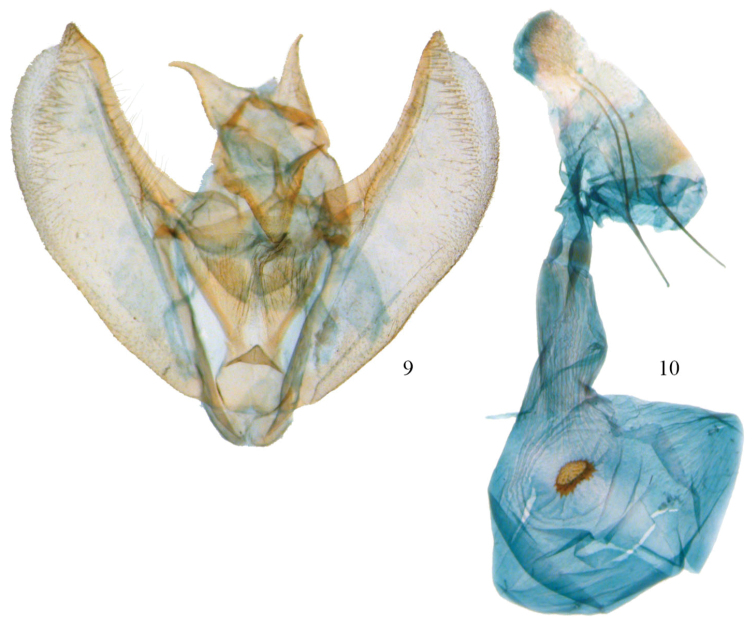
*Phyllodonta intermediata*. **9** male valve (JBS_3312) **10** female genitalia (JBS-5409).

**Male.** (Fig. 7) *Head*–labial palps warm brown, slightly porrect. First segment upcurved, second segment similar in length and tufted slightly over third segment, which is 1/3 as long and angled ventrally. Tongue developed. Eyes hemispherical, large, ocellus present. Frons brown, slightly pointed with scaling directed anteriorly. Scape cream proximally, brown laterally and distally. Cream color extends along dorsal edge of antennae to tip. Antennal segments oblong with minute setae at base of each segment. Interantennal area warm brown, lighter onto collar. *Thorax and Abdomen*–Vertex brown, fine scaling extending onto thorax, most scales expanding to three pointed tip, a few with black tips. Tegulae brown, abdomen with closely appressed scaling, gray brown dorsally, warm brown ventrally. Legs finely scaled, warm brown, tibia on first two legs with 3 evenly spaced white spots dorsally, proximal smallest, distal largest, tarsal spines prominent (0-2-4). *Wings*–forewings warm brown with prominent antemedial and postmedial lines that undulate, in both cases forming two outward bulges. Antemedial bulges separated by cleft. Both lines black edged with lighter grayish scales proximally. Black scaling distal to reniform at costa and forming a diffuse line paralleling antemedial line. Reniform spot small, black and forming center of a gray circle. Wings long, outer margin truncated at costa with a distinct notch medially, forewing length 23.1 mm (21–25 mm, n=10). Hindwing with prominent postmedial line, margin with submedial notch. Underside of wings warm brown, forewing with postmedial line prominent, medial line visible, antemedial line absent. White blotch subterminal at notch, larger black blotch proximal to it. Small discal spot present. Hindwing underside with prominent PM line, prominent discal spot, small yellow line of scales distal to PM line on both wings. Cream streak from wing base widening to anal angle. *Male Genitalia* (Figs 6, 9) (10 dissections) –uncus unsclerotized, short with terminal setae. Socius triangular shaped at base and curving distally toward tip. Outer margin often straight, but sometimes excurved basally. Well-defined tegumen with triangular gnathos, rounded at tip with small setae. Juxta narrow basally forming a pocket and widening toward transitilla lobes, which widen medially. Costa well sclerotized tapering to tip of valva. Valva broad with two ridges bearing setae. Subcostal ridge bears well-differentiated setae, tapers slightly and meets a broader submarginal ridge that broadens to half width at valve tip. Saccus vee-shaped and indented slightly at basal point. Aedeagus partially sclerotized distally, a band of striations at tip. Ductus inserts sub-basally. Vesica sac-like, curving ventrally; two outpockets medially, most prominent curving to right with smaller lobe to left. No cornuti or sclerotized areas. Vesica slightly longer than aedeagus. **Female.** (Fig. 8) – similar to male but larger, gray-brown ground color, cross lines usually more prominent, forewing length 24.3 mm (22.5–26.0, n=2). Gray circle around reniform distinct. Undersides similar to male but gray brown. *Female Genitalia* (Figs 10, 17) (2 dissections) – Anal papillae with setae, distally truncated in shape with anterior apophyses shorter than posterior apophyses. Anal plate not sclerotized, forming a broad funnel. Ductus bursae short with sclerotized collar at posterior end; collar 2× as broad as in other two species. Accessory bursae striated, widening significantly at midpoint forming a hump and weak striations continuing onto broadly rounded, almost triangular corpus bursae. Corpus bursae 2× as wide as ductus bursae, mostly unstriated with crescent-like signum. Signum somewhat variable, often peanut shaped with large, broad spines anteriorly and short spines posteriorly. Very similar to the signum found in *Phyllodonta esperanza* but always wider between anterior and posterior edges.

#### Barcodes.

Seven specimens have been barcoded and exhibit two haplotypes that differ from each other by a maximum of 0.5%. They differ from those of *Phyllodonta esperanza* by a minimum of 4.6% and from *Phyllodonta alajuela* by a minimum of 6.3%. The most common haplotype (11-CRBS-286) is:

AACATTATATTTTATTTTTGGAATTTGAGCTAGAATAGTGGGAACGTCTTTAAGTTTATTAATTCGAGCAGAATTAGGGAATCCTGGGTCTTTAATTGGAGATGATCAAATTTATAATACTATTGTAACTGCACATGCTTTTATTATAATTTTCTTTATAGTAATACCTATTATAATTGGGGGATTTGGAAATTGATTAATTCCTTTAATACTAGGGGCTCCTGATATAGCTTTCCCTCGAATAAATAATATAAGATTTTGGTTACTTCCACCTTCCATTACATTATTAATTTTTAGAAGAATTGTAGAAAATGGAGCTGGAACAGGATGAACAGTTTACCCACCTTTATCTTCTAATATTGCTCATGGGGGTAGTTCTGTTGATCTTGCTATTTTTTCATTACATTTAGCTGGTATTTCATCAATTTTAGGAGCTATTAATTTCATCACCACAATTATTAATATACGATTAAATAATTTATCTTTTGATCAAATACCTTTATTTGTATGAGCGGTAGGAATTACTGCATTTTTATTATTATTATCATTACCTGTTTTAGCTGGAGCTATTACTATATTATTAACCGATCGAAATTTAAATACATCTTTTTTTGACCCTGCTGGTGGAGGAGATCCAATTTTATACCAACATTTATTT

#### Distribution.

Known from between 1275 to 2280 m in the Talamancas, the Central Volcanic range, and the Tilaran range in Costa Rica. The moths are probably in flight throughout the year.

#### Remarks.

Nothing is known about the biology of this species. Its range may extend into the other mountain ranges in Costa Rican and perhaps into northern Panama.

### Phyllodonta alajuela

Taxon classificationAnimaliaLepidopteraGeometridae

Sullivan sp. n.

http://zoobank.org/F193BB75-F3BF-42EC-9292-C10D65E15331

Figs 11 –15 , 18

#### Type material.

Holotype male: Costa Rica, San Ramon Reserva Biol. Alberto M. Brenes Estacion Biol. (10.22°N, 85.62°W), Alajuela Province, 850 m, 7–11 February 2005, J. Bolling Sullivan (07-CRBS-365, JBS-3303) (INBio). Paratypes: 4♂, 1♀: 2♂, same data as holotype (JBS-5401, 5407), 1♂, 1♀, Costa Rica, Upata. Estacion San Gerardo (10.89°N, 85.38°W), Alajuela Province, 550 m, 17–21 July 2006, J. Bolling Sullivan (JBS-5410, 07-CRBS-366,1205 (barcoded twice), JBS-3314). 1♂, Costa Rica, Upata Bijagua, Alberque Heliconias (10.43°N, 85.01°W), 800 m, Alajuela Province, J. Bolling Sullivan (JBS-3310).

#### Etymology.

The species is named for the province where the holotype and paratypes were taken. It has also been taken in Guanacaste province.

#### Diagnosis.

Maculation does not seem to distinguish this species. It is best characterized by barcode data and the genitalia. To date it has been found below 1200 m and with no other member of the *Phyllodonta latrata* complex. In the male, the distal sides of the socii are usually straight and the vesica is armed with cornuti at the end of the most prominent pouch (distal side of socii swollen in *Phyllodonta esperanza*, both *Phyllodonta esperanza* and *Phyllodonta intermediata* have unarmed vesicas). The female has a broadened collar on the ductus bursae (narrow in *Phyllodonta esperanza*)and the signum on the corpus bursae is almost round with equal-sized spines around it (crescent shaped in *Phyllodonta esperanza*, peanut shaped in *Phyllodonta intermediata*).

#### Description.

**Figures 11, 12.**
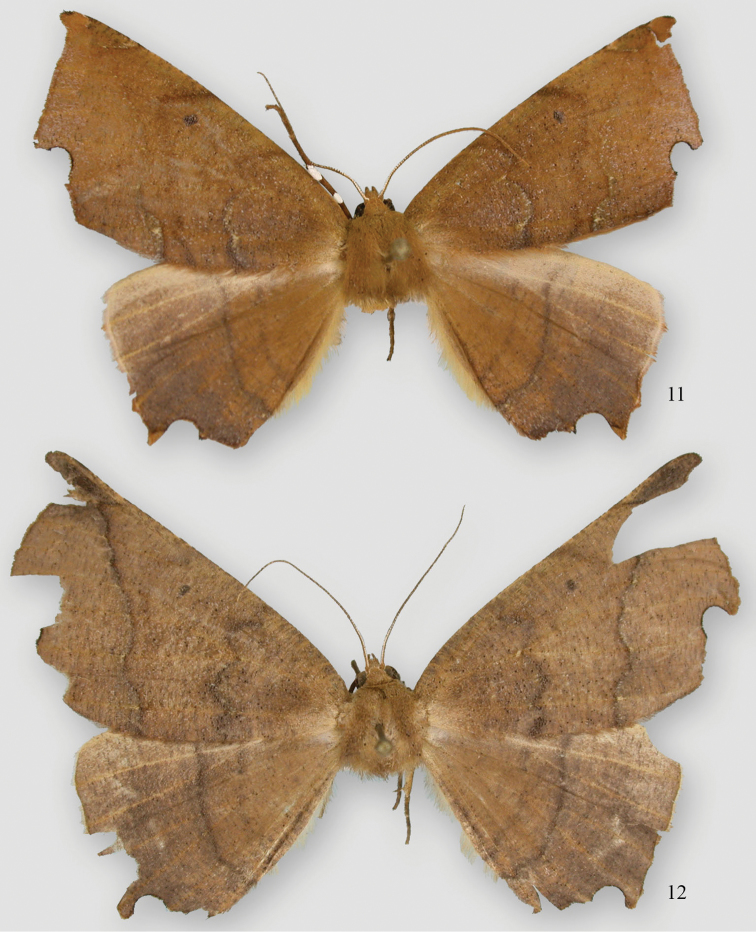
*Phyllodonta alajuela*. **11** male holotype (07-CRBS-365) **12** female (07-CRBS-366.

**Figures 13–15.**
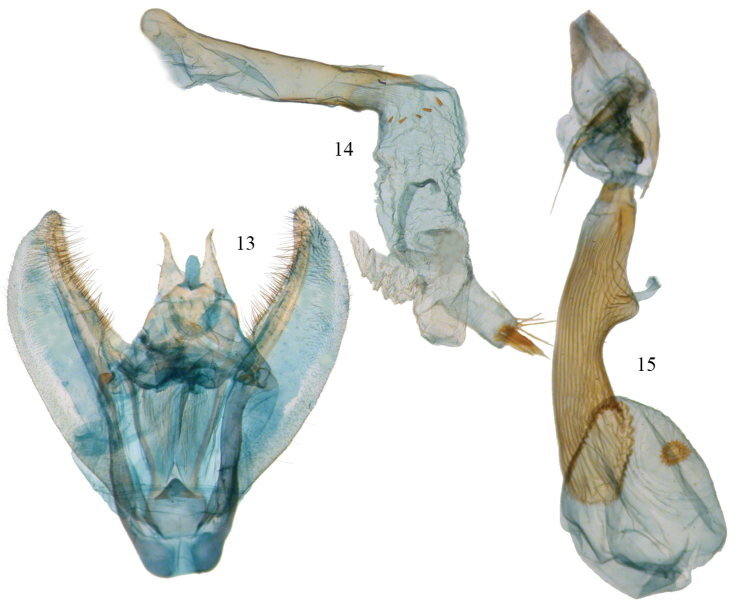
*Phyllodonta alajuela*. **13** male valve (JBS-5400) **14** aedeagus (JBS-3303) **15** female genitalia (JBS-3314).

**Male.** (Fig. 11) *Head*–labial palps warm brown, slightly porrect. First segment upcurved, second segment similar in length and tufted slightly over third segment, which is 1/3 as long and angled ventrally. Tongue developed. Eyes hemispherical, large, ocellus present. Frons brown, slightly pointed with scaling directed anteriorly. Scape cream proximally, brown laterally and distally. Cream color extends along dorsal edge of antennae to tip. Antennal segments oblong with minute setae at base of each segment. Interantennal area warm brown, lighter onto collar. *Thorax and Abdomen*–Vertex brown, fine scaling extending onto thorax, most scales expanding to three pointed tip, a few black tipped. Tegulae brown, abdomen with closely appressed scaling, gray brown dorsally, warm brown ventrally. Legs finely scaled, warm brown, tibia on first two legs with 3 evenly spaced white spots dorsally, proximal smallest, distal largest, tarsal spines prominent (0-2-4). *Wings*–forewings warm brown with prominent antemedial and postmedial lines that undulate, in both cases forming two outward bulges. Antemedial bulges separated by cleft. Both lines black edged with lighter grayish scales proximally. Black scaling distal to reniform at costa and forming a diffuse line paralleling antemedial line. Reniform spot small, black, and forming center of a gray circle. Wings long, outer margin truncated at costa with a distinct notch medially, forewing length 24.1 mm (23.5–25.0 mm, n=6). Hindwing with prominent postmedial line, margin with submedial notch. Underside of wings warm brown, forewing with postmedial line prominent, medial line visible, antemedial line absent. White blotch subterminal at notch, larger black blotch proximal to it. Small discal spot present. Hindwing underside with prominent PM line, prominent discal spot, small yellow line of scales distal to PM line on both wings. Cream streak from wing base widening to anal angle. *Male Genitalia* (Figs 13–14) (6 dissections) –uncus unsclerotized, over half length of socii with terminal setae. Socius triangular shaped not swollen at base and curving distally toward tip. Well-defined tegumen with triangular gnathos, rounded at tip with small setae. Juxta narrow basally forming a pocket and widening toward transitilla lobes, which widen medially. Costa well sclerotized tapering to tip of valva. Valva broad with two ridges bearing setae. Subcostal ridge well developed with prominent setae, tapering slightly and meeting a broader submarginal ridge that broadens to half width at valve tip. Saccus vee-shaped and usually indented slightly at basal point. Aedeagus partially sclerotized distally, a band of striations at tip. Ductus inserts sub-basally. Vesica tubular with two outpockets distally, most prominent from left side and terminating with a patch of 10 or more moderately long cornuti. A row of small cornuti at base of vesica and variable in appearance, sometimes absent. Vesica slightly longer than aedeagus. **Female.** (Fig. 12) – similar to male but larger, gray-brown ground color, cross lines usually more prominent, forewing length 27 mm, n=1. Gray circle around reniform more distinct. Undersides similar to male but more gray brown. *Female Genitalia* (Figs 15, 18) (1 dissection) – Anal papillae with setae, distally truncated in shape with anterior apophyses shorter than posterior apophyses. Anal plate not sclerotized, forming a broad funnel. Ductus bursae short with narrow sclerotized collar at posterior end; collar ends form obvious abutment. Accessory bursae striated, widening significantly to form nipple at midpoint and weakly defined striations continuing onto bursae. Corpus bursae almost round. Signum circular with two or three rows of short spines radiating from fovea.

**Figures 16–18.**
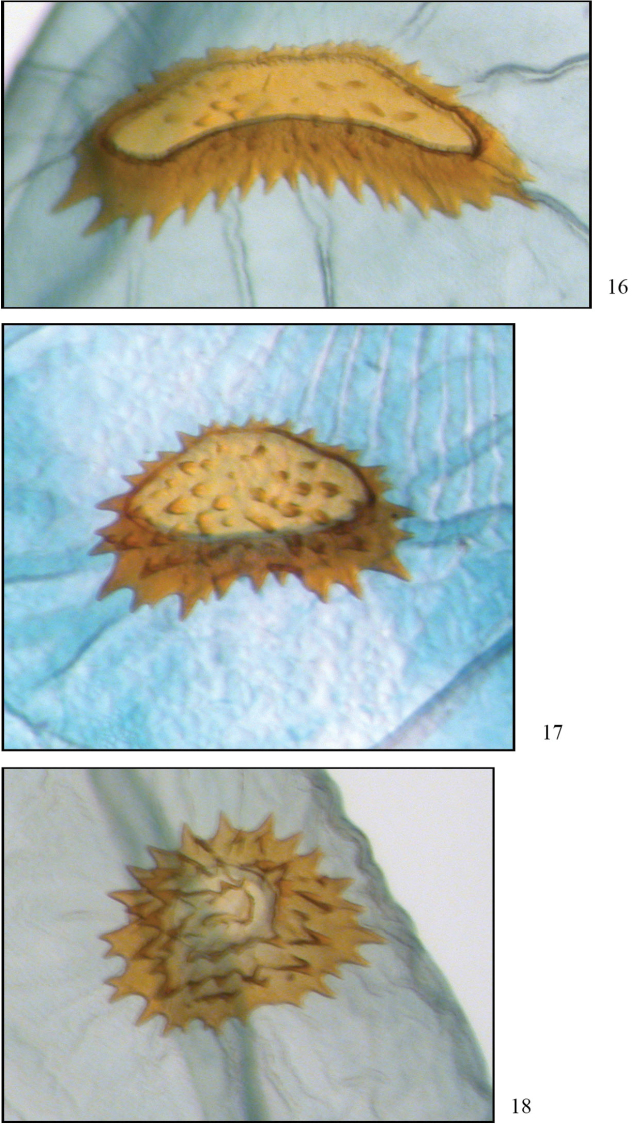
Comparison of signa. **16** *Phyllodonta esperanza* (JBS-3315) **17** *Phyllodonta intermediata* (JBS-5409) **18** *Phyllodonta alajuela* (JBS-3314).

#### Barcodes.

Twenty eight specimens have been barcoded and exhibit 9 haplotypes that differ from each other by a maximum of 1%. They differ from those of *Phyllodonta esperanza* by a minimum of 4.8% and from *Phyllodonta intermediata* by a minimum of 6.3%. The most common haplotype (09-SRNP-36297) is:

AACATTATATTTTATTTTTGGAATTTGAGCTGGAATAGTAGGTACATCTTTAAGTTTATTAATTCGAGCGGAATTAGGAAACCCTGGGTCTTTAATTGGAGATGATCAAATTTATAATACTATTGTAACTGCTCATGCTTTTATTATAATTTTTTTTATGGTAATACCTATTATAATTGGGGGATTTGGGAATTGATTAGTTCCTTTAATATTGGGGGCCCCAGATATAGCTTTCCCACGAATAAATAATATAAGATTTTGATTACTTCCGCCTTCTATTACACTTTTAATTTCTAGAAGAATTGTAGAAAATGGAGCCGGAACTGGATGAACTGTCTACCCTCCTTTATCTTCTAATATTGCCCACGGTGGTAGTTCTGTTGATCTTGCTATTTTTTCATTACATTTAGCTGGTATTTCATCAATTTTAGGGGCTATTAATTTTATTACAACAATTATTAATATACGATTAAATAACTTATCTTTTGATCAAATACCTTTATTTGTTTGAGCTGTAGGAATCACTGCATTTTTATTATTATTATCATTACCTGTTTTAGCTGGAGCTATTACTATATTATTAACTGATCGAAATTTAAATACATCTTTTTTTGACCCTGCTGGAGGAGGAGACCCAATTTTATATCAACATTTATTC

#### Distribution.

Known from 500 to 1150 m in the provinces of Alajuela and Guanacaste. Moths are probably in flight throughout the year.

#### Remarks.

Janzen and Hallwachs (2014) have reared this species 62 times. The larvae fed primarily (59 records) on *Witheringia solanacea* L. Hér. in the Solanaceae. One record may represent a second species on *Brugmansia × candida* (Solanaceae). The present range of *Phyllodonta alajuela* seems to be limited to the Northwestern provinces of Costa Rica and may extend into Nicaragua. Southward and eastward its range is unknown. Specimens of the *Phyllodonta latrata* complex from Anchicaya and Calima Dam in Valle Province of Colombia (four dissections) have similar male genitalia, but the basal spines on the vesica are larger; no specimens of it have been barcoded. Species of non-migratory moths common to Colombia and Costa Rica are often distributed in the western lowlands of both countries.

## Discussion

**Figures 19, 20.**
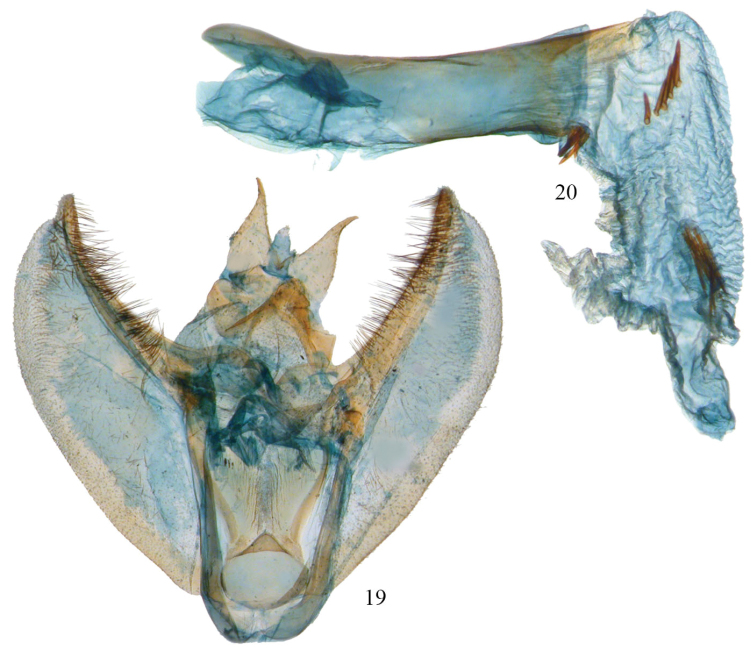
*Phyllodonta latrata* complex from Bahia State, Brazil (Camacan, 600 m, 2-III-1994, V. O. Becker) (USNM-50814). **19** male valve **20** aedeagus.

As currently applied, the *Phyllodonta latrata* (Gn.) complex is represented in Costa Rica by three new species that can be differentiated by barcodes and genitalic features. *Phyllodonta alajuela* occurs at lower altitudes whereas *Phyllodonta intermediata* and *Phyllodonta esperanza* overlap at intermediate altitudes; but are often separate at the altitude extremes of their ranges. At the conclusion of this study, two male specimens in the *Phyllodonta latrata* complex from the states of Bahia and Rio De Janeiro in Brazil were borrowed from the USNM and dissected. Both dissections showed characters not seen in any other members of the complex (Figs 19–20) and differed from each other as well.

The complex nature of species grouped under the name *Phyllodonta latrata* is not uncommon among mid and high elevation moth species in the neotropics (Sullivan 2011). Constant change in weather over long periods of time, and the rise and fall of mountains in the region, have provided optimal conditions for speciation. The use of barcoding provides a valuable tool for measuring species delineation and it is only when barcoding has segregated individuals of the apparent complexes that one can find reliable structural characters to sort the species. It is very difficult to sort normal interspecific variation in maculation and genitalic characters from intraspecific variation when species are closely related. If speciation is recent or ongoing, such characters may not be discernable but in the case of the *Phyllodonta latrata* complex in Costa Rica such characters are apparent. The arrangement of the pouches emanating from the vesicas of the males and the shapes of the signa in the females are key structures (with their changes perhaps related) and are supported by other less obvious characters and geographic separation.

Barcodes and genitalic analyses also support the differentiation of other populations of the *Phyllodonta latrata* complex in South America. Specimens from lowland western Colombia may be conspecific with *Phyllodonta alajuela* but proof is lacking. Two male specimens taken above 2500 m in the central Andean range (Alto Rio Quindio) were dissected and differed from each other and all species from Costa Rica. Two specimens from coastal Brazil also were unique. Additional specimens barcoded from Ecuador and Peru are distinct. Thus, it is likely that the type locality for *Phyllodonta latrata* (Brazil) and high altitude locations throughout the Andes will provide a surprising number of additional species in the complex.

## Supplementary Material

XML Treatment for Phyllodonta esperanza

XML Treatment for Phyllodonta intermediata

XML Treatment for Phyllodonta alajuela
